# In-Frame Amber Stop Codon Replacement Mutagenesis for the Directed Evolution of Proteins Containing Non-Canonical Amino Acids: Identification of Residues Open to Bio-Orthogonal Modification

**DOI:** 10.1371/journal.pone.0127504

**Published:** 2015-05-26

**Authors:** James A. J. Arpino, Amy J. Baldwin, Adam R. McGarrity, Eric M. Tippmann, D. Dafydd Jones

**Affiliations:** 1 School of Biosciences, Main Building, Park Place, Cardiff University, Cardiff, CF10 3AT, United Kingdom; 2 Department of Chemistry, Indiana–Purdue University Fort Wayne, Fort Wayne, Indiana, 46815, United States of America; Berlin Institute of Technology, GERMANY

## Abstract

Expanded genetic code approaches are a powerful means to add new and useful chemistry to proteins at defined residues positions. One such use is the introduction of non-biological reactive chemical handles for site-specific biocompatible orthogonal conjugation of proteins. Due to our currently limited information on the impact of non-canonical amino acids (nAAs) on the protein structure-function relationship, rational protein engineering is a “hit and miss” approach to selecting suitable sites. Furthermore, dogma suggests surface exposed native residues should be the primary focus for introducing new conjugation chemistry. Here we describe a directed evolution approach to introduce and select for in-frame codon replacement to facilitate engineering proteins with nAAs. To demonstrate the approach, the commonly reprogrammed amber stop codon (TAG) was randomly introduced in-frame in two different proteins: the bionanotechnologically important cyt *b*_562_ and therapeutic protein KGF. The target protein is linked at the gene level to sfGFP via a TEV protease site. In absence of a nAA, an in-frame TAG will terminate translation resulting in a non-fluorescent cell phenotype. In the presence of a nAA, TAG will encode for nAA incorporation so instilling a green fluorescence phenotype on *E*. *coli*. The presence of endogenously expressed TEV proteases separates *in vivo* target protein from its fusion to sfGFP if expressed as a soluble fusion product. Using this approach, we incorporated an azide reactive handle and identified residue positions amenable to conjugation with a fluorescence dye via strain-promoted azide-alkyne cycloaddition (SPAAC). Interestingly, best positions for efficient conjugation via SPAAC were residues whose native side chain were buried through analysis of their determined 3D structures and thus may not have been chosen through rational protein engineering. Molecular modeling suggests these buried native residues could become partially exposed on substitution to the azide containing nAA.

## Introduction

The advent of expanded genetic code approaches has allowed proteins to be engineered to contain new chemistry not normally present in the natural amino acid repertoire (see [1–3] for recent reviews). Through the use of reprogrammed codons or entirely new codon systems in conjunction with engineered translation machinery, upwards of 100 non-canonical amino acids (nAAs) can be incorporated into a wide range of cell types (from bacteria to mammalian) and even whole organisms (e.g. *Caenorhabditis elegans* [4], *Drosophila melanogaster* [5] and *Arabidopsis thaliana* [6]). Reprogrammed genetic code systems essentially makes any member of the proteome, whether native or recombinant, accessible through defined and targeted nAA incorporation during cellular protein synthesis. Furthermore, as proteins containing nAA become more valuable in a commercial context, recombinant expression can generate high yields of protein product for downstream applications [7, 8].

The most common expanded genetic code approach is to reprogramme the low usage amber (TAG) stop codon [9, 10]. Reprogramming is implemented using engineered tRNA-amino-acyl/tRNA synthase pairs that incorporate a desired nAA in response to the UAG codon during cellular protein synthesis [2]. This approach has recently been further enhanced through the development of new *E*. *coli* strains with all native TAG stop codons removed from the genome along with it associated ribosomal release factor RF1 [11].

While protein engineering using nAAs is becoming more established it still suffers from the drawback of traditional rational site-directed mutagenesis: limited understanding of the effect of a nAA on protein structure and function leading to the use of a fairly rudimentary design processes. As has been repeatedly observed during rational protein engineering (see reference [12, 13] for two excellent recent examples), the impact of nAA incorporation at a particular site may not always be obvious, and while our understanding of the effect of a particular nAA on the protein folding-structure-function relationship is increasing [14–16] it is still very limited. Directed evolution (see [17–19] for a sample of available reviews) was developed as a concept to address the shortcomings in rational protein engineering by taking a Darwinian approach through the generation of molecular diversity followed by library screening and selection. By sampling the whole protein backbone variants with new or enhanced properties containing non-intuitive mutations are routinely isolated.

**Fig 1:**
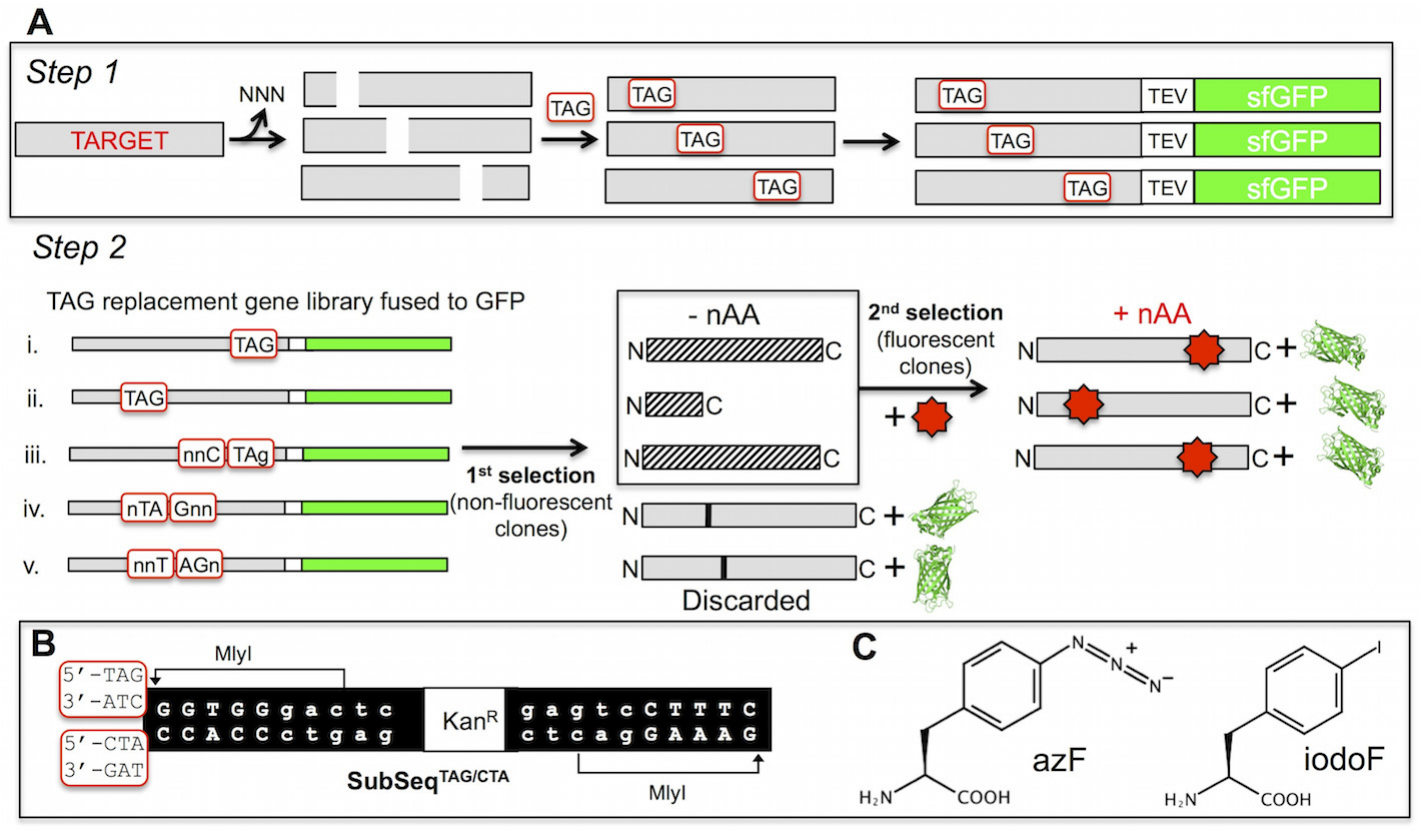
In-frame TAG codon replacement. (A) *Step 1* involves trinucleotide deletion followed by TAG donation using a combination of the engineered transposon MuDel [25] and the DNA cassette SubSeq [21] essentially as described previously [23] and outlined in B. The TAG substitution library is then cloned in front of the TEV-sfGFP cassette in plasmid pIFtag (S1 Fig). *Step 2* outlines the selection for in-frame TAG substitutions. Initially, cells are grown in the absence of a nAA and non-fluorescent colonies selected; fluorescent colonies are removed at this stage as they are deemed not to have an in-frame TAG due to the generation of a full translation product. The second selection involves plating the selected colonies in the presence of nAA. Those cells that regain fluorescence suppress TAG termination due to nAA incorporation and thus produce sfGFP. (B) Alternate versions of the new SubSeq DNA cassette for donating TAG. The two alternatives are shown in the red boxes at the far left. (C) The two nAAs used in this study, *p*-azido-L-phenylalanine (azF) and *p*-iodo-L-phenylalanine (iodoF).

Directed evolution approaches to widen nAA sampling were until recently restricted to sampling defined regions [20]. The advent of transposon-based approaches that allowed random trinucleotide/codon (TriNEx) mutations events across the length of a gene [21, 22] opened up the opportunity for whole gene TAG sampling [23, 24]. This has proved useful in identifying residues in proteins such as GFP not previously sampled by traditional mutagenesis by which nAAs can modulate function [15]. The main problem with the basic approach was the quality of the libraries generated due to the inherent nature of the method. Trinucleotide exchange makes no allowance for codon positioning thus replacement may occur across two adjacent codons generating an out of frame TAG replacement (Fig 1A). Additionally, the cassette donating the TAG codon may insert in the wrong direction resulting in CTA codon insertion. In some instances CTA codon sample may be useful in expanding sampling by rescuing some cross codon replacement events where the last nucleotide of a codon is G (e.g. aaC TAg).

One important area that would benefit from combining nAA incorporation and broad protein sampling is defined and bioorthogonal protein conjugation [1, 26]. Attachment of chemically useful adducts to proteins is important for applications ranging from bioimaging to therapeutics. Traditionally, attachment has relied on utilising the inherent chemistry in a protein, normally amine, carboxyl or thiol groups. However, these groups are ubiquitous both within an individual protein and the proteome as whole leading to non-specific, non-optimal and in the case of complex biological mixtures targetless labelling. Thus the ability incorporate a reactive handle into a desired position in protein without any native biological reactivity is of great utility. Click chemistry [27], especially strain-promoted azide-alkyne cycloaddition (SPAAC) [28, 29] is becoming of particular interest due to it speed and compatibility with biological systems. Incorporation of the phenyl azide Click handle into a protein via the nAA *p*-azido-L-phenylalanine (azF) [30] makes Click chemistry a reality both *in vivo* and *in situ* [31]. However, little is known about the influence of residue microenvironment on azide reactivity in SPAAC in terms of efficiency and kinetics. Recent work suggests that the link between surface accessibility of the azide group and SPAAC is not straight forward and counter intuitive [32].

The challenge of trinucleotide exchange is to quickly identify desired TAG mutations without extensive sequencing and functional screening. Here we present a general approach that allows the generation of in-frame TAG codons across a gene of interest. The method utilises read-through of the target gene to superfolder GFP (sfGFP) [33] in the presence of a nAA. In the absence of nAA, the TAG reverts to a stop codon so prematurely stopping translation in the target gene before reaching the sfGFP segment. A TEV protease cleavage site is present between the target and sfGFP so that the activity of the two proteins can be decoupled soon after production through endogenously expressed TEV. We demonstrate the approach using two proteins, cytochrome *b*_562_ (cyt *b*_562_) and keratinocyte growth factor (KGF). Modification by SPAAC with a fluorescent dye was achievable for only certain cyt *b*_562_ residues that were buried in the native, non-mutagenized structure.

## Materials and Methods

### Materials

AzF and iodoF were purchased from Bachem and dissolved in 0.25 M NaOH prior to use. DBCO-585 was purchased from Click Chemistry Tools and dissolved in DMSO to a stock concentration of 2.5 mM.

### Fusion plasmid construction

**Fig 2:**
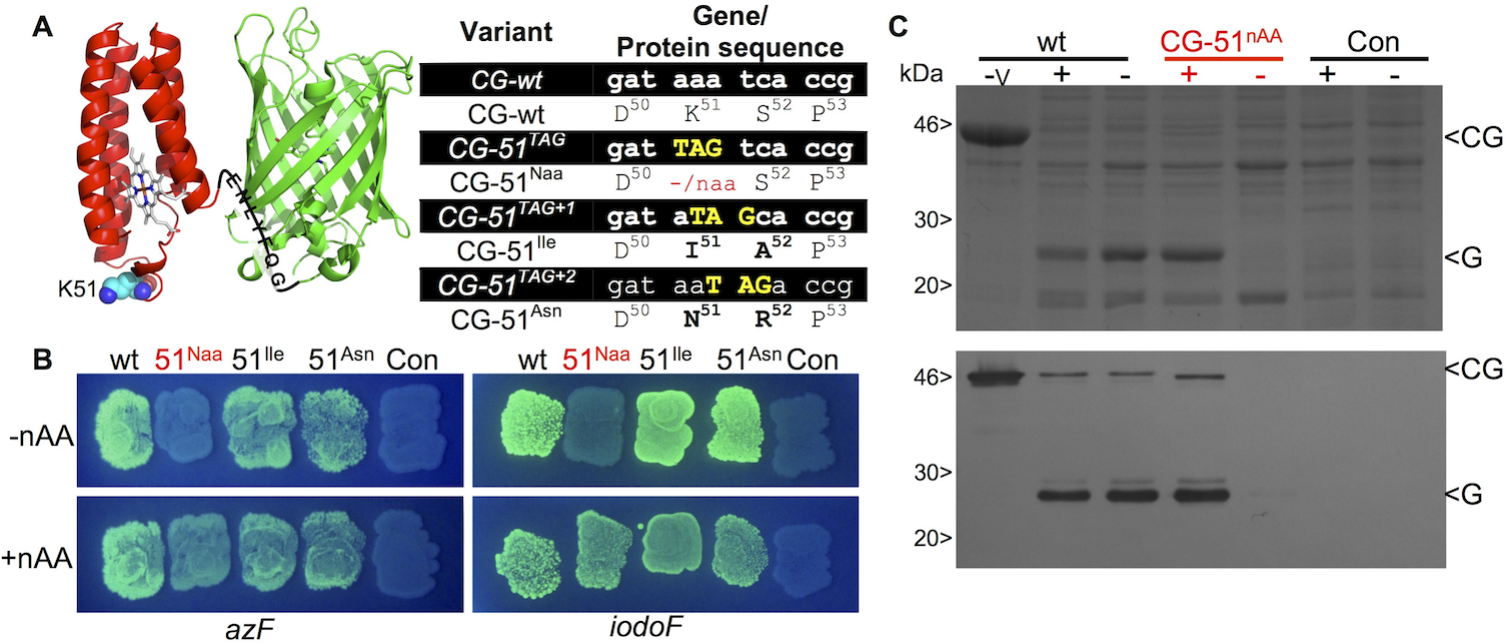
Selection for in-frame TAG replacement. (A) Schematic of the cytochrome (red) and sfGFP (green) fusion with the TEV cleavage site between the two shown. The residue targeted (K51) for replacement and the rational TAG replacements are shown. (B) cellular fluorescence of each variant in the absence (-nAA) and presence (+nAA) of either azF or iodoF. The wt refers to the cyt *b*_562_-sfGFP fusion without any TAG codon replacement. (C) SDS-PAGE (top panel) and Western blot (bottom panel) analysis of fusion protein expression in the soluble fraction of the cell lysate. The—v lane represents cells producing CG-wt in the absence of the pAB vector. The + and—lanes refer to the presence or absence of the nAA, respectively. CG and G refer to the cyt *b*_562_-sfGFP fusion (~40 kDa) and the sfGFP alone (27 kDa) generated through TEV cleavage. The Western blot detection was performed with an antiGFP primary antibody.

The pET-22b(+) vector (Novagen/Merck) formed the basis for constructing pIFtag, with the gene of interest expressed as a fusion with sfGFP under control of the T7 promoter. The two fused proteins are separated via a TEV protease sequence (Fig 2B and S1 Fig). In this study either cyt *b*_562_ or KGF were used as the N-terminal fusions to sfGFP. This plasmid was initially constructed by splicing PCR amplified fragments containing either the mature portion of the *cybC* gene (primers 109/112) or *sfGFP* (primers 104/105) via a linker encoding a new XhoI recognition site and the TEV cleavage site. NdeI and SalI sites were added to the 5’ and 3’ ends of the fragment, respectively. The resulting fragment was ligated into the NdeI/XhoI sites in pET-22b(+), thereby destroying the XhoI site within pET-22b(+), but adding an additional XhoI site 5’ of the TEV cleavage site. Alternative genes of interest can be easily sub-cloned into this vector upstream of the TEV site and sfGFP gene using NdeI/XhoI.

The 3 mutants, cg-51^TAG^/51^Naa^, *cg-51* ^*TAG+1*^/CG-51^Ile^ and *cg-51* ^*TAG+2*^/CG-51^Asn^, whereby a TAG codon was either added in-frame or +1 or +2 nucleotides with respect to K51 was achieved by site-directed mutagenesis. Primer pairs 092/093, 092/102 and 092/103 were used to generate each respective TAG mutation. The mutations were confirmed by DNA sequencing. All primers sequences are listed in S1 Table.

### Western blotting

*E*. *coli* Tuner cells hosting the pAB and pIFtag were grown in LB broth supplemented with 100 μg/ml ampicillin and 34 μg/ml chloramphenicol. The pAB plasmid harbours the iodoF aminoacyl-tRNA-synthetase (aaRS^iodoF^) and two tRNA^CUA^ required for nAA incorporation during cellular protein synthesis (S1 Fig). The aaRS^iodoF^ is promiscuous and can be used for high efficiency and fidelity incorporation of both iodoF and azF [15]. Protein production was induced with 100 μM IPTG, and culture medium supplemented with 5 mM p-iodoPhe. Cells were then incubated at 37°C with shaking overnight. Cells were collected and resuspended at an OD_600_ of 10 in BugBuster (1 ml), 200 μg/ml lysozyme and benzonase (0.5 μl/1 ml BugBuster) and incubated at room temperature for 30 min. Following fractionation by centrifugation (20 min, 16000 *g*, 4°C) 10 μl of soluble fraction was analysed on a 15% SDS-PAGE alongside NEB prestained protein marker. Proteins were blotted and detected with anti-GFP antibody (1:10,000) and 2° antibody (1:10,000). Detection was performed using the BioRad Immun-Blot AP Colorimetric Kit.

### Library construction: cyt *b*_562_

The initial cyt *b*_562_ library was constructed in the plasmid pNOM [25] plasmid essentially as described previously [21, 23]. Introduction of the MuDel transposon throughout the pNOM:*cybC* plasmid was performed using MuA and generated ~4.3x10^4^ cfu on transforming *E*. *coli*. The colonies were pooled and plasmid DNA isolated. Replacement of MuDel by SubSeq^TAG/CTA^ in the vector was achieved by digestion of the plasmid pool with MlyI to remove MuDel and 3 bp from the host plasmid, followed by dephosphorylation, purification following agarose gel electrophoresis, and ligation with the SubSeq^TAG^ or SubSeq^CTA^ cassette DNA. Transformation of *E*. *coli* DH5α resulted in ~2.3 x10^4^ CFU, with ~55% incorporating SubSeq^TAG^ (~1.3x10^4^ CFU) and 45% SubSeq^CTA^ (1.0x10^4^ CFU), as calculated from individual transformations. The colonies were pooled and a second digestion with MlyI removed SubSeq. Intramolecular ligation of digested vector gave ~8.7 x10^4^ CFU following transformation of *E*. *coli* DH5α.

The *cybC* ^TAG^ library was sub-cloned into a modified version of pIFtag (S1 Fig) between the NcoI and XhoI sites upstream of encoded TEV cleavage site and sfGFP. Modified pIFtag has the more amenable NcoI restriction site in place of NdeI but is otherwise identical to that in S1 Fig. It was necessary to use PCR on this occasion to add the relevant 5’ NcoI recognition sequence. The library was amplified using Phusion High-Fidelity DNA Polymerase and primers 131 and 111 in 16 separate tubes for 20 cycles before subcloning into modified pIFtag.

Transformation of *E*. *coli* DH5α gave ~5.0x10^3^ CFU, which were pooled and plamid DNA isolated. The pooled pIFtag DNA was then used to transform electrocompetent *E*. *coli* Tuner cells already containing pAB (S1 Fig). Colony fluorescence was determined using a transilluminator. Non-fluorescent colonies (192 selected at random) were transferred to 96-well microtitre plates and replica plated onto rectangular agar plates containing chloramphenicol (34 μg/ml), carbenicillin (100 μg/ml), IPTG (100 μM) and iodoF (2 mM). After 24 hrs the plates were moved to 4°C. Colonies that became fluorescent in the presence of iodoF were sequenced. All sequenced variants contained in-frame TAG codons and variants covered ~15% of the *cybC* gene.

### Library construction: KGF

DNA encoding codon-optimized keratinocyte growth factor (*kgf*) lacking a termination codon was subcloned from pBMH (Biomatik) to pNOM-XP6 [34, 35] via NcoI and XhoI sites. Transposition of MuDel into pNOM-XP6:*kgf* using MuA for either 4 hours or overnight yielded approx. 2.0x10^4^ cfu after transformation into *E*. *coli* BL21 Gold (DE3) electrocompetent cells. Following transformation, cells were plated on LB agar containing chloramphenicol (34 μg/ml) and cells scrapped from the plate and pooled. DNA was purified from the pooled cells and digested with NcoI and XhoI. The band corresponding to MuDel incorporated within the *kgf* gene (~1.7 kb) was isolated and ligated with pNOM-XP6 also digested with NcoI/XhoI. The resulting sample was used to transform *E*. *coli* BL21 Gold and yielded 1.3 x10^4^ cfu. MuDel was removed from *kgf* using MlyI and replaced by SubSeq^TAG^ and SubSeq^CTA^. Subsequent ligation and transformation resulted in 2.4 x10^4^ variants selected on kanamycin containing LB agar plates. SubSeq was excised via MlyI digestion and the remaining vector was recircularised via intramolecular ligation. Subsequent transformation of *E*. *coli* DH5α resulted in more than 1.5 x10^6^ cfu when selected on LB agar plates supplemented with 100 μg/ml carbenicillin. The resulting library DNA was digested with NcoI/XhoI and subcloned into modified pIFtag. *E*. *coli* BL21 Gold (DE3) cells containing pAB were transformed and plated on LB agar plates containing 100 μg/ml ampicillin, 34 μg/ml chloramphenicol and 200 μM IPTG. A total of 77 non-fluorescent colonies were transferred to 96-well format and replica plated onto LB agar containing 100 μg/ml carbenicillin, 34 μg/ml chloramphenicol and 2 uM IPTG plus 2 mM azF, and incubated at 30°C overnight. The sequence of 16 colonies that were non-fluorescent in the absence of nAA and fluorescent in the presence were determined.

### Protein production

*E*. *coli* BL21 Gold (DE3) cells were used to produce cyt *b*_562_ or KGF incorporating azF in response to the amber (TAG) stop codon using a reprogrammed genetic code system similar to that described previously [23, 36]. Cells were co-transformed with the relevant pIFtag plasmid housing either the cyt *b*_562_ or KGF gene and pAB. Single colonies grown on ampicillin and chloramphenicol LB agar were used to inoculate 1 mL LB broth cultures supplemented with the same antibiotics. After 6 hours of growth at 37°C, the 1 mL cultures were used to inoculate 10 mL LB supplemented with ampicillin and chloramphenicol. Cultures were grown at 37°C with shaking until an OD_600_ of 0.4–0.8 was achieved then 2.5 mM azF was added and the cultures grown for a further hour. Protein expression was induced by the addition of 500 μM IPTG for cyt *b*_562_-TAG variants or 100 μM IPTG for KGF-TAG variants and incubated at 37°C for 24 hours. Cells were harvested by centrifugation and lysed in 50 mM Tris-HCl, pH 8.0, 150 mM NaCl containing 0.2 mg/mL lysozyme, 1 mM PMSF and 1 mM EDTA by sonication.

### Strain-Promoted Azide-Alkyne Cycloaddition (SPAAC)

SPAAC reactions were performed, as described elsewhere [32], using crude cell lysates (to mimic intracellular conditions more closely) as the source of azF-Cyt *b*_562_ or azF-KGF. SPAAC was performed using cell lysates from 5 mL expression cultures standardised to an A_600_ of 1. Reactions were performed with 5 μM of DBCO-585 at 25°C for 24 hours.

### Fluorescent SDS-PAGE Analysis

SPAAC reactions were analysed using fluorescent imaging after SDS-PAGE. The reaction components were separated by 20% SDS-PAGE using established protocols. Gel bands were imaged and analysed using a Typhoon 9400 Variable Mode Imager with a 532 nm excitation laser and a 610 nm emission filter with a 30 nm band pass. Images were processed using ImageJ software. SDS-PAGE gels were subsequently stained with Coomassie Blue stain (50% (v/v) methanol, 10% (v/v) acetic acid, 0.1% (w/v) R250 Coomassie blue) and imaged to view all proteins.

### *In silico* modelling of azF containing cyt *b*_562_ variants

Structure files for iron protoporphyrin IX were made using Avogadro [37]. Force field parameters were derived based on the electrostatic model approach, which uses electrostatic potential (ESP) to describe the electronic structure of the porphyrin [38]. Geometry optimization and ESP calculations were performed using GAMESS-US [39] at the HF/6-31G* level in order to be consistent with the AMBER99sb force field [40]. The published crystal structure for holo-cyt *b*_562_ (PDB code: 256B [41]) was used as the starting point for simulation of both proteins. For the P45azF cyt *b*_562_ variant the published structure for apo-cyt *b*_562_ (PDB code: 1APC [42]) was used instead. The structures were altered using a python script within MacPyMol to mutate the chosen residue to azF prior to energy minimisation, which was carried out using the GROMACS software package [43]. Molecular dynamic simulations were carried out using the AMBER99sb force field, modified with the parameters for iron protoporphyrin IX and azF. The starting structure was placed within a triclinic box with dimension of 6.4x6.1x7.8 nm. This was populated using the SPC water model to solvate the system to a total number of 16986 solvent molecules. The system was first energy minimised by performing 500 steps of steepest descent method followed by 500 steps of Conjugant Gradient method. The lowest energy state of the system was used as the starting conformation for the molecular dynamics simulation. The simulations were conducted at a constant temperature and pressure of 300 K and 1 atm (NPT). A cutoff of 8Å was chosen for nonbonded interactions and long range electrostatic interactions were characterised using the Particle Mesh Ewald (PME) [44]. The simulations were carried out for a total of 2 ns with a time step of 2 fs and the trajectories were analysed using various GROMACS components and visualised using VMD [45] (www.ks.uiuc.edu/Research/vmd/).

## Results and Discussion

### In-frame TAG selection process

The general approach for selecting in-frame TAG codons required for nAA incorporation is outlined in Fig 1A. TAG replacement in a target gene using the TriNEx process has been described previously [23]. However, there was no easy way to determine in-frame and correctly orientated TAG codons. To address this problem and to make the method more general in its application, a new plasmid system was constructed (S1 Fig). The vector pIFtag housed the sfGFP gene within the cloning site to allow direct gene fusion downstream of the target protein bridged by a TEV protease sequence. The pAB vector was based on pAA [46] but housed two additional elements. The first is a gene encoding inducible and endogenous production of TEV protease to allow for *in situ* cleavage of the target protein-sfGFP fusion to prevent any potential adverse downstream effects of the fusion event. The second is the promiscuous *p*-iodo-L-phenylalanine (iodoF) aminoacyl tRNA synthetase under the control of the promoter and terminator from the endogenously expressed glnS gene (*E*.*coli* glutaminyl-tRNA synthetase). This iodoF aminoacyl tRNA synthetase is known to efficiently encode incorporation of various nAAs [15]. To maximise the number of clones with in-frame TAG codons, a new version of the TAG donating DNA cassette (SubSeq [21, 23]) was generated which sampled the complement sequence (CTA) in case of reverse cassette insertion (Fig 1B).

By generating the TAG codon replacement library (Fig 1A, step 1) and placing it in the context of pIFtag (and accompanied by pAB) allows selection due to nAA incorporation based on cellular green fluorescence (Fig 1A, step 2). The initial phase is to remove any cross codon exchange events during trinucleotide exchange that may generate amino acid substitution mutations by colony selection in the absence of nAA. Variants with an in-frame TAG codon will generate truncated products within the target and no sfGFP. Out-of-frame TAG events will generate substitutions leading to translational read through to sfGFP resulting in cellular fluorescence. Non-fluorescent colonies are retained for the second selection round in which the presence of the nAA will result in its incorporation at the position dictated by the TAG codon. Translational read through from the target gene to sfGFP will occur resulting in cellular fluorescence. Variants that were non-fluorescent in the first selection round due to other undesirable mutational events will thus also be removed. While we have used colony picking to separate fluorescence and non-fluorescent cells, automated and high throughput systems such as FACS can be used to facilitate the process.

To test that the system can select for in-frame TAG codons, a set of site-directed mutants were generated in the small electron transfer protein, cytochrome *b*_562_ (cyt *b*_562_; Fig 2A). Cytochrome *b*_562_ is proving to be an important system for studying metalloprotein electron transfer at the single molecule level, and as a novel molecular electronic [47–49], biosensing [50, 51] and controlled assembly scaffold [52–55]. To facilitate its use for both fundamental and applicative studies, optimal interfacing with non-biological materials is required, which will be aided by the use of nAAs with suitable coupling chemistry. For example, incorporation of *p*-azido-L-phenylalanine (azF; Fig 1D) can introduce new photochemical cross-linking and bioorthogonal conjugation properties onto a target protein [31].

Cyt *b*_562_ placed in-frame with sfGFP and the two linked via the TEV protease cleavage sequence (Fig 2A) resulted in cells displaying an obvious green fluorescence phenotype (Fig 2B). Replacement of the codon for Lys51 with TAG (*cg-51* ^*TAG*^/CG-51^nAA^) gave a nAA-dependent cell fluorescence phenotype; cellular fluorescence was only observed in the presence of a nAA in the culture medium. Shifting the TAG codon +1 (*cg-51* ^*TAG+1*^/CG-51^Ile^) or +2 (*cg-51* ^*TAG+2*^/CG-51^Asn^) nucleotides with respect to K51 did not instill a nAA-dependent green fluorescence phenotype (Fig 2B); despite the native amino acid substitution mutations in cyt *b*_562_, read through to sfGFP occurred as predicted. Both SDS-PAGE and Western blot confirmed that protein was only produced in the presence of nAA for the in-frame TAG variant and that cellular production of TEV protease gave rise to *in situ* cleavage of the sfGFP from cyt *b*_562_ (Fig 2C).

The transposon-based random TAG codon replacement approach was applied to cyt *b*_562_. The exact details of each step used to generate the library are outlined in the Methods section. Sequencing revealed that all the colonies with a green fluorescence phenotype only in the presence of iodoF had an in-frame TAG (Table 1). A total of 16 different variants were observed spread across cyt *b*_562_. The observed mutations sampled a wide variety of structural and functional aspects of cyt *b*_562_, including each helical element and their linking loops, residues with different degrees of surface exposure and vicinity to the heme centre (Table 1). The observed frequency of several variants was high (e.g. A29nAA & P56nAA) but is in keeping with previous work concerning the use of the MuDel transposon system [21, 25, 34]. While MuDel inserted across the whole breadth of a target gene, there are thought to be preferred insertion sequences based on a consensus sequence, previously reported to be NPyG/CPuN [56]. Based on sequencing generated from the data derived from this current study and libraries generated as part of previous published work [50, 57] (S2 Fig) there does not appear to be an absolute consensus sequence, but there does appear to be a higher proportion of G/C rich pentamer sequences. This suggests that MuDel may have a higher target site preference for G/C rich stretches of DNA. The nature of the TAG codon replacement event varied as expected between true codon and cross codon replacement. Only 4 variants could be categorically ascribed to a cross codon event with only one resulting in a substitution mutation in an adjacent amino acid; the other three resulted in silent mutations (Table 1).

**Table 1:** Sequence and characteristics of observed cyt *b*_562_ TAG replacement variants.

cyt *b*_562_ mutation	Nucleotide change	Freq	Apo SASA (Å^2^) ^a^	Holo SASA (Å^2^) ^a^	Secondary structure ^c^
K19N,A20nAA	aa**a-gc**g → aa**C-TAg**	1	9 b	17 b	Lp H1-H2
A20nAA	aaa-**gcg** → aaa-**TAG**	3	9 b	17 b	Lp H1-H2
A24nAA	gc**g-gc**g → gc**C**-**TA**g	3	81 e	59 e	H2
A29nAA	gac-**gcg** → ga**c-TAg**	12	3 b	1 b	H2
P45nAA	acg-**ccg** → acg-**TAG**	6	19 b	4 b	Lp H1-3_10_ ^b^
S52nAA	aaa-**tca** → aaa-**TAG**	2	25 b	58 e	Lp 3_10_–H3
P53nAA	tca-**ccg** → tca-**TAG**	8	100 e	104 e	Lp 3_10_–H3
P56nAA	agc-**ccg** → ag**c-TAg**	12	82 e	109 e	H3
L68nAA	at**t-ct**g → at**C-Ta**g	2	68 e	14 b	H3
Q71nAA	gg**t-c**ag → gg**C-TA**g	6	78 e	39 pe	H3
A75nAA	ga**c-gcg** → ga**c-TAg**	2	22 pe	9 b	H3
L78nAA	aag-**ctg** → aag-**TAG**	4	17 pe	37 pe	H3
K83nAA	ggt-**aaa** → ggt-**TAG**	1	115 e	97 e	Lp H3-H4
Q88nAA	gcg-**cag** → gcg-**TAG**	1	84 e	31 pe	H4
A91nAA	gct-**gca** → gct-**TAG**	3	46 pe	2 b	H4
Q103nAA	ca**c-cag** → ca**c-TAg**	1	143 e	36 pe	H4

^a^ calculated solvent accessible surface area (SASA) using GETAREA (http://curie.utmb.edu/getarea.html) b, buried; pe, partially exposed; e, exposed.

^b^ residue interacting with heme.

^c^ H1, H2, H3 and H4 refers to helices 1, 2, 3 and 4 running from the N- to C-terminus, with Lp referring to the corresponding linking loops.

### Bioconjugation of cyt b_562_ by SPAAC

**Fig 3:**
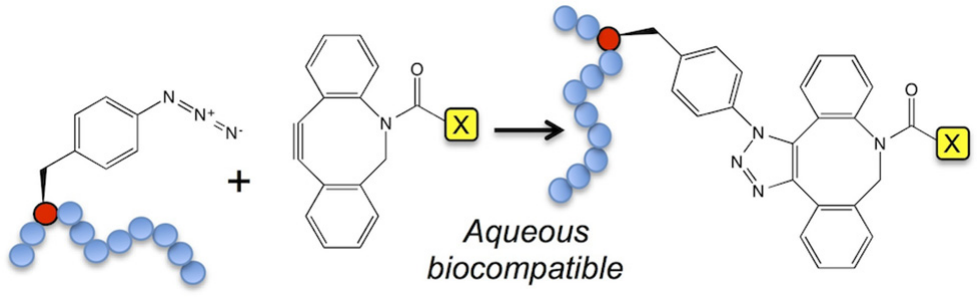
SPAAC between genetically encoded azide (red sphere) within a protein (blue spheres) and an activate alkyne (dibenzylcyclooctyne;DBCO). A triazole link is formed between the azide and alkyne groups.

**Fig 4:**
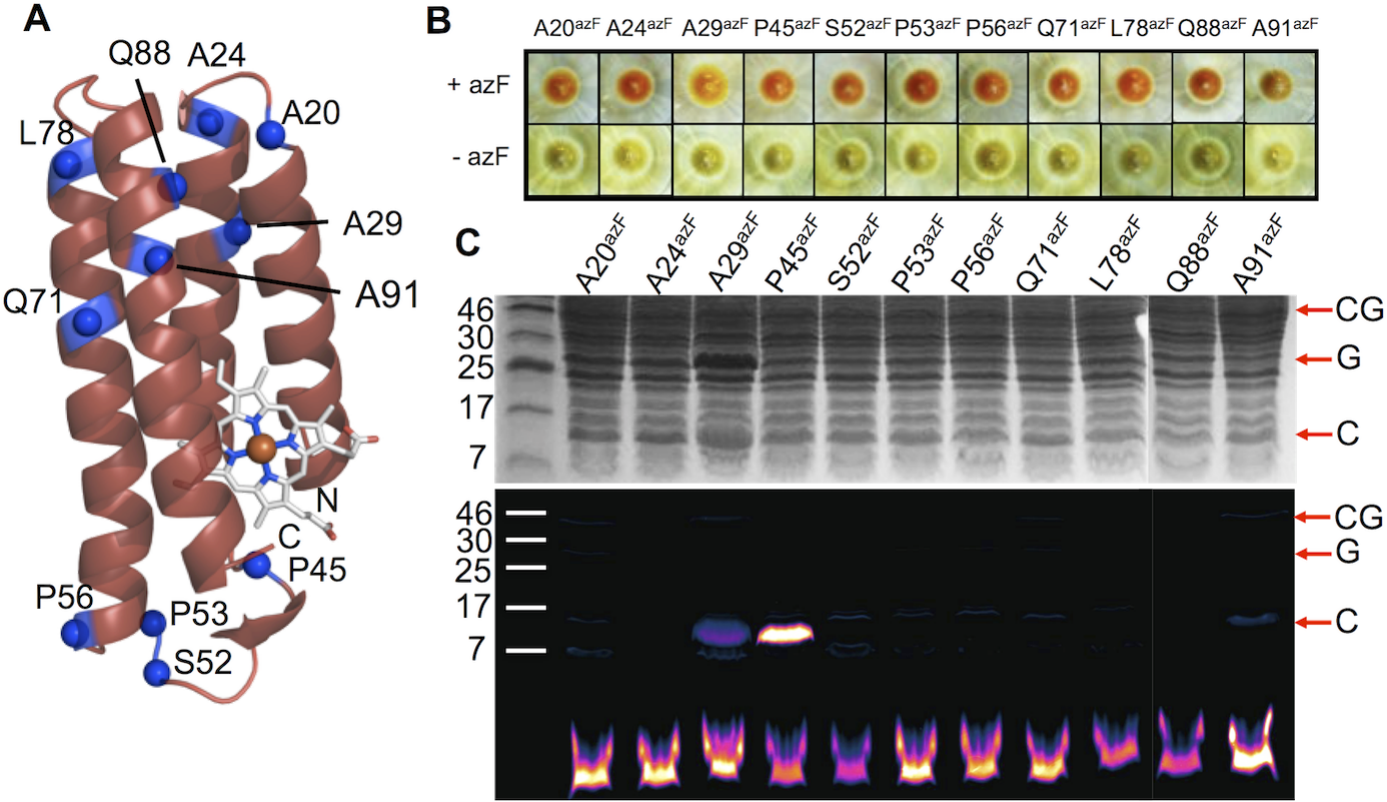
Production and conjugation of cyt *b*_562_ azF-containing variants. (A) Schematic structure of cyt *b*_562_ showing the residues substituted with azF. (B) Expression of cyt *b*_562_ variants housed in the pIFtag plasmid in the presence (+azF) or absence (-azF) of azF. Cells successfully expressing functional cyt *b*_562_ display a red phenotype. (C) SDS-PAGE analysis of SPAAC conjugation. The top panel shows the Coomassie Blue stained and the bottom is fluorescence imaging of the DBCO-585 moiety. CG, G and C refer to cyt *b*_562_-sfGFP fusion, sfGFP and cyt *b*_562_, respectively. The lower band is unreacted DBCO-585. For the sake of transparency, the samples Q88 and A91 were run on separate gels to the others and the resulting images electronically linked to the other samples.

The SPAAC reaction is outlined in Fig 3. Essentially, the azF acts as the protein embedded azide component that can react orthogonally under biologically compatible conditions with an activated alkyne (cyclooctyne) [29]. As the azide group is prerequisite for SPAAC reactions and not present in any natural biomolecule, modification with a derivative harbouring an alkyne (C≡C) will be specific to defined residue position within the target protein amongst the cellular milieu or *in vitro*. Several variants identified during TAG codon screening were assessed for the reactivity of the introduced phenyl azide group to its alkyne partner (Fig 4A) that sampled a wide variety of cyt *b*_562_ structural features. Dibenzylcyclooctyne (DBCO) housing the strained ring alkyne group linked to the fluorescence rhodamine dye Texas red (here on in termed DBCO-585) was used to test for residue conjugation ability and efficiency [32].

The 11 variants selected for further analysis were successfully expressed in the presence of azF as indicated by the red-pink phenotype due to the production of full length holo cyt *b*_562_ (Fig 4B); little or no expression was apparent in the absence azF. The colour of cells expressing two variants, A29^azF^ and A91^azF^, were different to the others; the A29^AzF^ cell pellet was more orange and A91^azF^ brown. Both A29 and A91 lie close to each other in the protein close at the opposite end of the protein from the haem binding pocket (Fig 4A). SDS-PAGE revealed that TEV protease cleavage *in situ* was largely complete leaving free cyt *b*_562_ separate from sfGFP (Fig 4C). One variant, A29^AzF^, appeared to be produced at a higher level than the others, as indicated by the darker band equivalent to sfGFP (~27 kDa) and cyt *b*_562_ (~13 kDa).

To assess how placement within the protein affected the reactivity of the phenyl azide group, conjugation with DBCO-585 was performed. Only two variants, A29^azF^ and P45^azF^, were modified to any extent with the DBCO-585 derivative in cell lysates (Fig 4C). The native residues at both these positions are buried in the determined structures of apo and holo forms, with P45 being close to the heme co-factor in the holo-form (Table 1). The higher apparent brightness of the P45^azF^ cyt *b*_562_ variant suggests it was modified to a greater extent even though more A29^azF^ cyt *b*_562_ protein was produced (Fig 4C). The maintenance of a coloured phenotype suggests that both variants still retain the capacity to bind haem on azF incorporation.

There does not appear to a simple correlation between residue position encoding azF, its relative solvent accessibility of the native residue, position in secondary structure and the ability to undergo SPAAC when replaced by azF. Most of the observed azF containing cyt *b*_562_ variants involve replacement of an exposed or a partially exposed residue (in apo or holo cyt *b*_562_). Overexpressed engineered cyt *b*_562_ is routinely produced as a mixture of apo and holo protein [58], both of which are produced as soluble proteins, so it is pertinent to take into account both these forms. Three residues replaced with azF have their original residues buried in both apo and holo forms according to the determined structures (Table 1), two of which (A29 and P45) are the only ones open to conjugation via SPAAC. A24 resides in the same helix (H2) as A29 with both residues separated by just over one helical turn and occupying similar positions in both the apo and holo cyt *b*_562_ structures. However, the A24 residue is exposed to the solvent while A29 is buried: only when azF is incorporated at residue 29 can cyt *b*_562_ be modified. This suggests that on replacement of A29 with the bulkier and longer azF, the side chain must at least become partially accessible to the solvent in either the apo and/or holo form. In an attempt to rationalise the effect of A29^azF^ mutation, the variant was modelled *in silico* using the holo-cyt *b*_562_ structure as a template. The putative molecular model of holo-cyt *b*_562_ [41, 59] A29^azF^ revealed that the phenyl azide may potentially exert a significant local structural effect and the reactive azide component may become surface exposed (Fig 4A). Rather than the phenyl azide pointing directly out to the solvent, the azide group may still be closely associated with the protein surface. Thus the modelling suggests replacement of the buried A29 with azF results in the critical reactive azide group becoming surface exposed without full exposure of the side chain as a whole.

**Fig 5:**
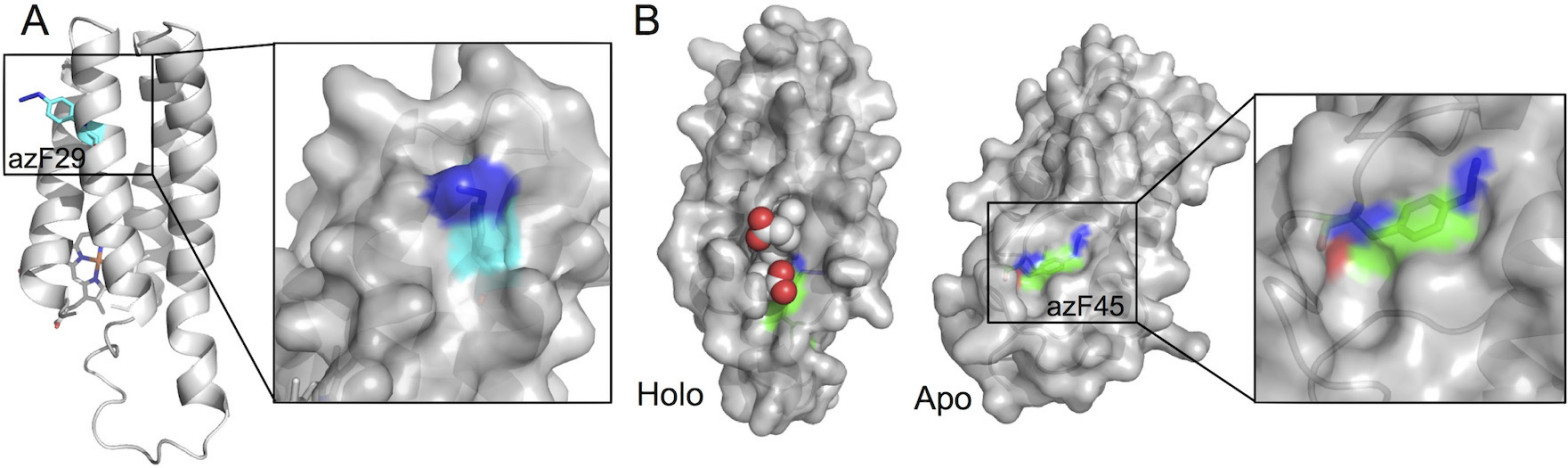
*In silico* modelling of azF cyt *b*_562_ variants. (A) Models of the A29^azF^ holo cyt *b*_562_. The mutated residue, azF29, is coloured in cyan at its carbon atoms. The right hand panel is a close up surface view of the model. (B) Models of the P45^azF^ apo and holo cyt *b*_562_ variant. The mutated residue, azF45, is coloured green at its carbon atoms. Haem is represented as spheres. The models were based on the PDB coordinate files 265B (Holo) and 1APU (apo). The figures where generated using PyMol [61].

A similar modelling approach was taken to rationalise the effect of the P45azF. The putative model of holo cyt *b*_562_ P45^azF^ suggested that haem may block accessibility for the incoming DBCO group (Fig 4B). The structure of apo-cyt *b*_562_ is known to be less structured around the C-terminal helix H4 [42, 60], making accurate modelling more difficult. Nonetheless, given that over-expressed cyt *b*_562_ is known to be a mix of holo and apo forms [58] modelling of the P45azF based on the available NMR structure of apo protein was attempted. The putative apo cyt *b*_562_ model of the P45^azF^ variant suggested that the azide group becomes surface accessible and thus available for modification (Fig 5B). As with the A29^azF^ model, the azide group and the phenyl azide side chain as a whole remains closely associated with the protein surface. Such a close surface association of the azide group was associated with the residues displaying the highest modification efficiency by SPAAC in sfGFP suggesting that a fully protruding phenyl azide may not be ideal [32]. Therefore, a key observation, at least in terms of SPAAC modification of protein embedded phenyl azides, is that burial of the native residue should not be overlooked as potential useful modification site and that positions likely to retain a degree of surface association on conversion to azF may be the ideal in terms of selecting high efficiency modification sites. Given that Click chemistry approaches like SPAAC are becoming more and more utilised, these observations may have wider implications in terms of the design of modification sites.

### Application of TAG in-frame mutagenesis to KGF

One of area of significant potential is the development of protein-based biopharmaceuticals through the defined and controlled modification with polymers and other useful agents [8, 62]. Derivatising a protein with large chemical adducts such as PEG shields them from proteolytic degradation and rapid removal from the body resulting in improved pharmacokinetics. However, most of the current approaches rely either on the inherent chemistry of the natural 20 amino acids (e.g. amine, carboxyl and sulfhydryl) or the introduction of terminal “modification” tagging sequences. Such lack of specificity in terms of position within the protein can give rise to unwanted heterogeneity of the modified protein sample and the site of modification may not be optimal for maximising protein stability/activity. One way of overcoming such problems is through the defined placement of nAAs with useful bioorthogonal and biocompatible reactivity, such as azF [31].

**Fig 6:**
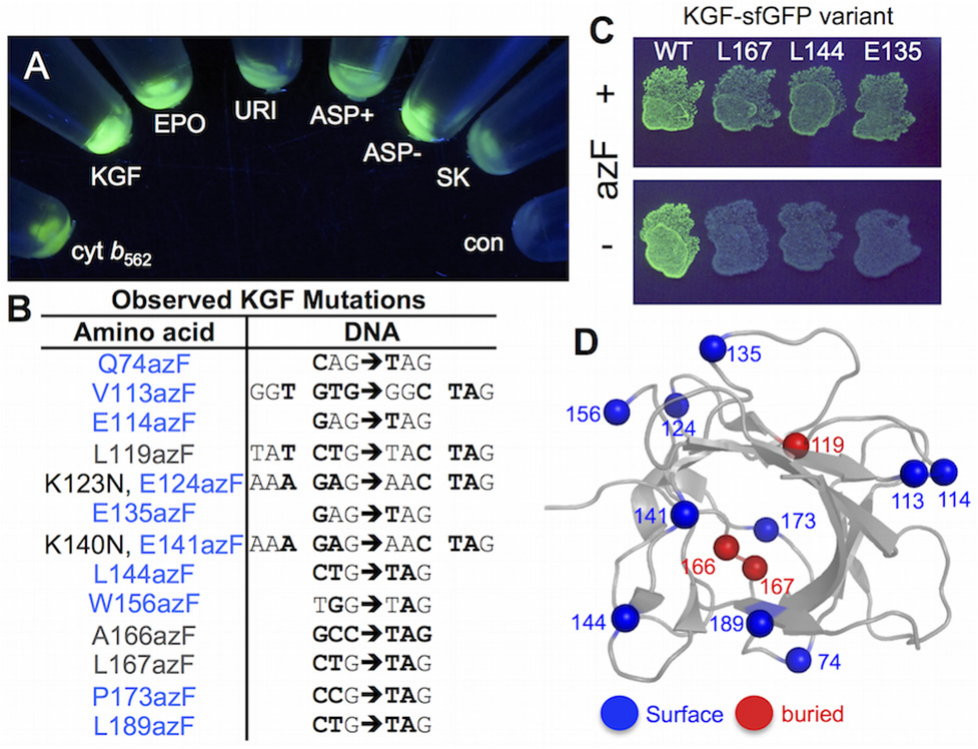
In-frame TAG replacement of KGF. (A) Compatibly of various therapeutic proteins with the *E*. *coli* sfGFP fusion screening approach. KGF, keratinocyte growth factor fragment; EPO, ethropoietin; URI, Uricase; ASP+ Asparaginase with N-terminal signal pepetide sequence; ASP- Asparaginase lacking N-terminal signal peptide sequence; SK, Streptokinase. The ‘con’ sample is the control cell sample. The proteins shown are all fused to sfGFP via the TEV digestion sequence. (B) Sequence of observed KGF variants. (C) azF-dependent expression of selected KGF-sfGFP variants. (D) Map of observed mutations on the structure of KGF, including an indication of whether the native residue is surface exposed (blue spheres) or buried (grey spheres) based on surface accessibility.

Initially, a range of different non-antibody therapeutic proteins were screened for their suitability for use with the sfGFP fusion library selection approach: (1) Keratinocyte growth factor (KGF) fragment (Kepivance); (2) Ethropoietin (EPO); (3) Uricase (URI; Rasburicase); (4) Asparaginase (ASP; Oncaspar); (5) Streptokinase (SK; Streptase). When cloned in-frame with sfGFP all the selected proteins conferred a fluorescence phenotype on *E*. *coli* (Fig 6A) suggesting that they will be compatible with the library screening approach. As a test for the system, library construction was focused on KGF as it was relatively small (139 amino acids) and conferred the brightest green fluorescent phenotype on *E*. *coli* under our expression conditions. The pharmaceutical version of KGF used here (sequence provided in S3 Fig), also known as Palifermin [63], is a recombinant analogue of human KGF with the first 23 residues at the N-terminal removed to improve stability. It stimulates keratinocyte growth in a variety of tissues and aids patient recovery from chemotherapy.

Using a combination of the two phase negative (non-fluorescent clones in absence of azF) and positive (fluorescent clones in the presence of azF) selection procedure, 13 different variants at the amino acid level were identified (Fig 6B). The requirement of the nAA for cellular gene fluorescence was confirmed by culturing cells expressing variants in the presence and absence of azF, three of which are show in Fig 6C as representatives. Only in the presence of azF was a fluorescence phenotype observed. Mapping the observed residues onto the known structure of KGF (PDB 1QQK;[64]) revealed that all but 3 (L119azF, A166azF, L167azF) of the variants were equivalent to exchange of surface exposed residues for azF (Fig 6D). While the current dogma suggests that surface exposure is critical as we have seen here and previously [32], this is not a guarantee for efficient modification by SPAAC especially when the mutation may result in a residue converting from buried to partially exposed. Thus, we did not ignore their potential for modification by SPAAC at this stage.

**Fig 7:**
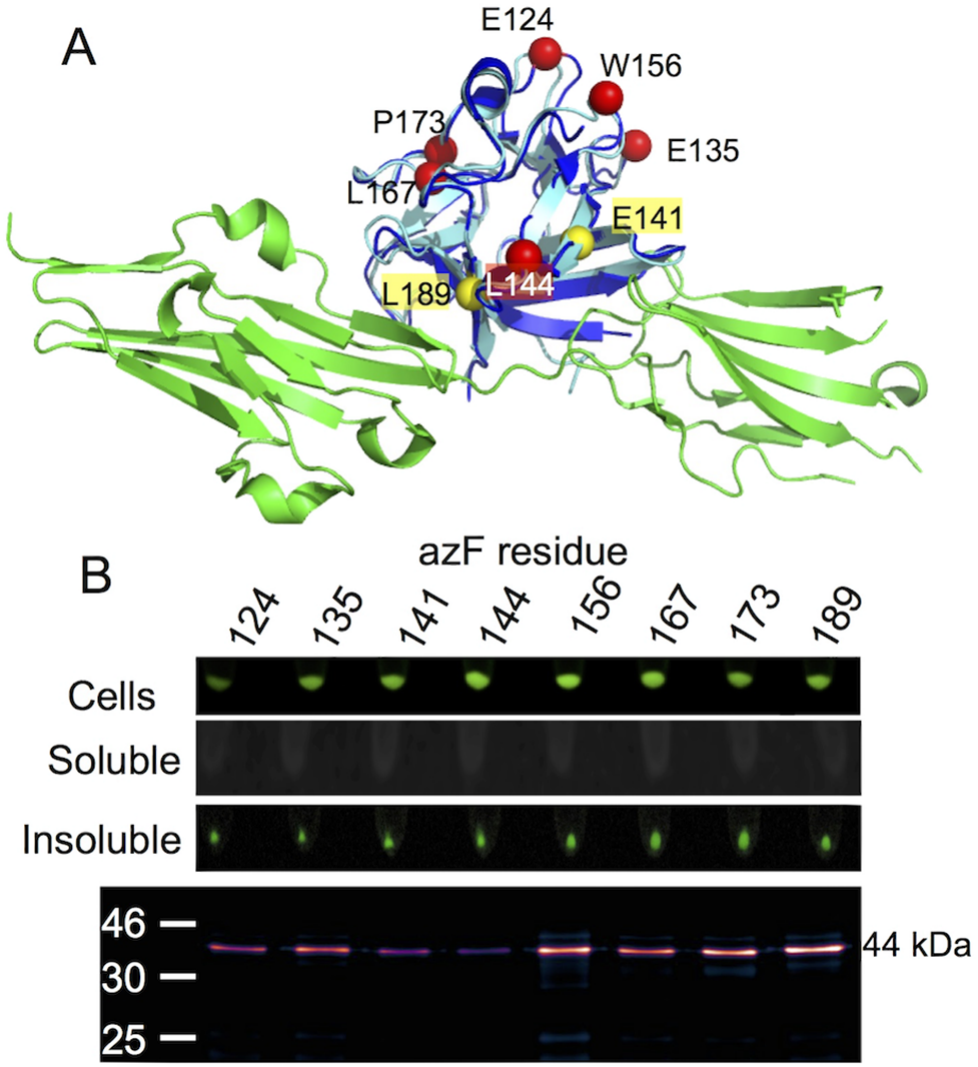
Production and SPAAC modification of KGF variants. (A) Superimposition of the KGF structure on the homologous FGF10-FGFR complex. Yellow and red spheres represent residues contributing to or not involved in the receptor interface. (B) Inherent sfGFP fluorescence of different cellular fractions (top two panels) and SPAAC modification with DBCO-585 of each analysed variant.

Another important requirement is that both mutation and the subsequent modification will not impede function by, for example, disrupting a key protein-protein interaction. KGF interacts with its cognate receptor FGFR2 and triggers various signalling pathways that simulate production of proteins required for cell growth and survival in epithelial cells [65]. There is currently no structure available for KGF bound to its cognate receptor FGFR2 so the KGF was modelled against the structurally analogous FGF10-FGFR complex (Fig 7A; 1EV2[66]). Analysis of the model revealed that six variants incorporate azF at positions distant from the protein-protein interface: E124, E135, L144, W156, L167 and P173. These variants together with two others with azF incorporated at E141 and L189 located at the receptor interface were chosen to assess their modification with DBCO-585 by SPAAC (Fig 7).

While attachment of sfGFP is supposed to aid solubility of attached proteins [33], all the KGF fusion variants were expressed as insoluble fusion proteins (Fig 7B and S4 Fig). While whole cells expressing the KGF-sfGFP were fluorescent suggesting that sfGFP matured (and thus folded) correctly, all the fluorescence was observed in the insoluble pellet fraction after cell lysis. The product size (~44 kDa) indicates that the insoluble KGF-sfGFP fusion was not cleaved by TEV suggesting the requirement for a soluble product for successful cleavage. This is in contrast to the cyt *b*_562_ fusions that produced largely soluble protein fusions that were essentially fully cleaved (Figs 2C and 3C). Despite insolubility and lack of fusion cleavage all eight KGF-sfGFP variants were capable of SPAAC modification with DBCO-585. Apart from 2 (E141^azF^ and L144^azF^), each KGF-sfGFP fusion variant was modified to a similar extent (Fig 7B). This is contrast to cyt *b*_562_ were only selected residues were modified. The insolubility coupled with likely aggregation may in the case of KGF facilitate general modification. This may be through, for example, the generation of local surface exposed hydrophobic regions in which a population of azide groups may reside; local hydrophobic patches have been suggested to aid SPAAC modification with DBCO [32], especially as the DBCO moiety is itself hydrophobic.

## Conclusion

We have shown here a general method for introducing in-frame amber stop codons for the directed evolution of proteins containing non-canonical amino acids. The link through to sfGFP expression and fluorescence allows identification of in-frame TAG codon replacements, and the *in situ* cleavage of predominantly soluble fusion products via TEV protease decouples sfGFP from the target protein. While we have demonstrated codon replacement with TAG, the method can be adapted for any of the emerging approaches for nAA incorporation (e.g. quadruplet codon systems [67, 68]) through changing the nature of the donating sequence in the SubSeq element. The ability to modify proteins in a truly bioorthogonal and biocompatible manner via SPAAC (and other nAA-dependent approaches [1]) has obvious benefits for both *in situ* and *in vitro* modification of proteins for fundamental and technological purposes. However, as we have shown here and elsewhere [32] simply choosing a surface exposed residues based on available structural information does not guarantee high efficiency modification. If this is the case, then a new strategy for protein engineers may be available for use with azF or other conjugation compatible nAAs and directed evolution may prove a fruitful manner by which to identify residues amenable to these next generation protein modification approaches.

## Supporting Information

S1 Fig Plasmids maps of (A) pIFtag and (B) the companion plasmid pAB. The pIFtag plasmid is based on pET22 except that downstream of the standard NcoI/NdeI and XhoI cloning site is the DNA sequencing encoding TEV digestion site and sfGFP. Cloning of a target gene (here with the cytochrome *b*_562_ sequence as an example) between the NcoI and XhoI site will put it in the same reading frame as the downstream elements. (B) The companion plasmid pAB is based on pAA reported previously as stated in the main text. It contains the engineered aminoacyl tRNA synthetase and tRNA for nAA incorporation as well as the T5 promoter driven expression of TEV protease.
(TIFF)

S2 Fig MuDel target site sequence analysis. A graphical representation of the frequency at which nucleotides appear at one of the 5 positions of the target site duplication, introduced during MuDel transposition. The graphical representation was produced using the WebLogo application (http://weblogo.threeplusone.com) from 181 unambiguous unique sequences sampled by MuDel from the TAG replacement library reported here together with TND and domain insertion libraries reported previously [69, 70]. The height of each simple at each of the 5 positions is relative to the observed frequency of a particular base.
(TIFF)

S3 Fig DNA (lower case) and protein (upper case) sequence of KGF used in this study. (TIFF)

S4 Fig SDS PAGE analysis of the soluble and insoluble cell lysate fractions of the KGF-sfGFP azF fusion variants. The band labelled K-G at ~44 kDa corresponds to the uncleaved KGF-sfGFP fusion product. All samples were standardised to set cell density (OD at 600 nm of 1.0).
(TIFF)

S1 Table Oligonucleotide primer sequences. (DOCX)
