# Supplementary figures and images for: In-Frame Amber Stop Codon Replacement Mutagenesis for the Directed Evolution of Proteins Containing Non-Canonical Amino Acids: Identification of Residues Open to Bio-Orthogonal Modification
Source: PLoS One. 2015 May 26;10(5):e0127504. doi: 10.1371/journal.pone.0127504 (PMC4444182; doi:10.1371/journal.pone.0127504)

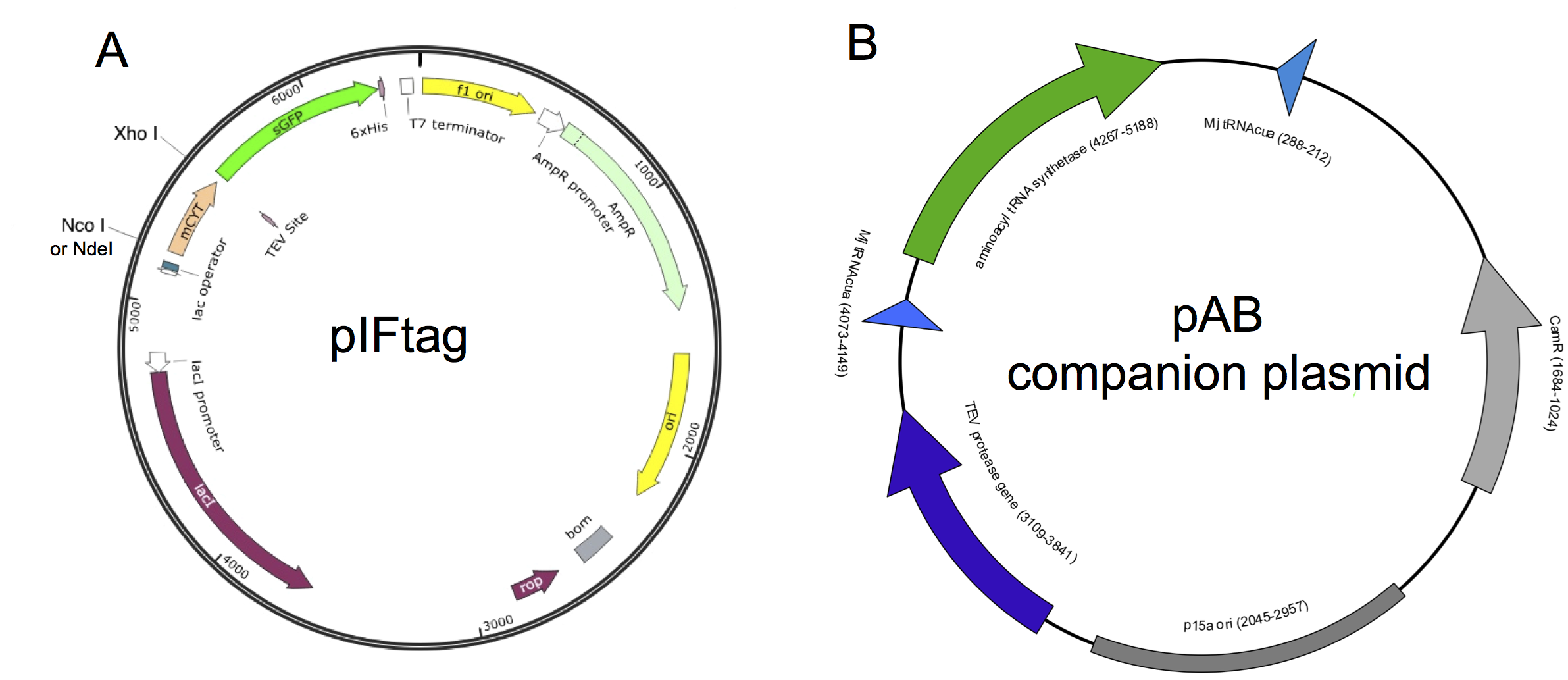

Supplement: S1 Fig — The pIFtag plasmid is based on pET22 except that downstream of the standard NcoI/NdeI and XhoI cloning site is the DNA sequencing encoding TEV digestion site and sfGFP. Cloning of a target gene (here with the cytochrome b 562 sequence as an example) between the NcoI and XhoI site will put it in the same reading frame as the downstream elements. (B) The companion plasmid pAB is based on pAA reported previously as stated in the main text. It contains the engineered aminoacyl tRNA synthetase and tRNA for nAA incorporation as well as the T5 promoter driven expression of TEV protease. (TIFF) [file pone.0127504.s001.tiff]

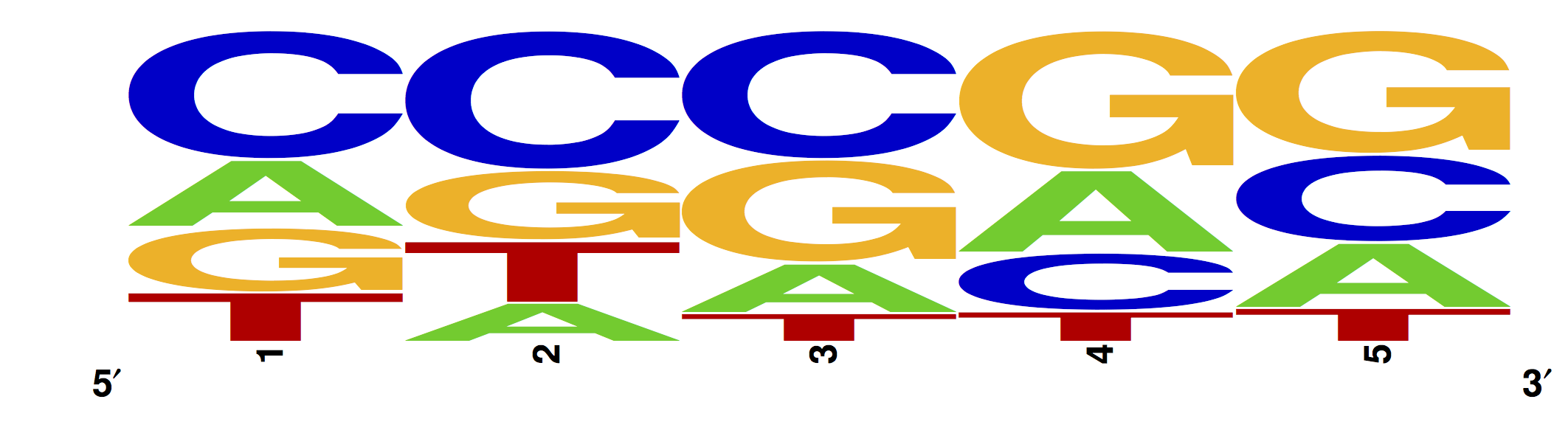

Supplement: S2 Fig — A graphical representation of the frequency at which nucleotides appear at one of the 5 positions of the target site duplication, introduced during MuDel transposition. The graphical representation was produced using the WebLogo application (http://weblogo.threeplusone.com) from 181 unambiguous unique sequences sampled by MuDel from the TAG replacement library reported here together with TND and domain insertion libraries reported previously [69, 70]. The height of each simple at each of the 5 positions is relative to the observed frequency of a particular base. (TIFF) [file pone.0127504.s002.tiff]

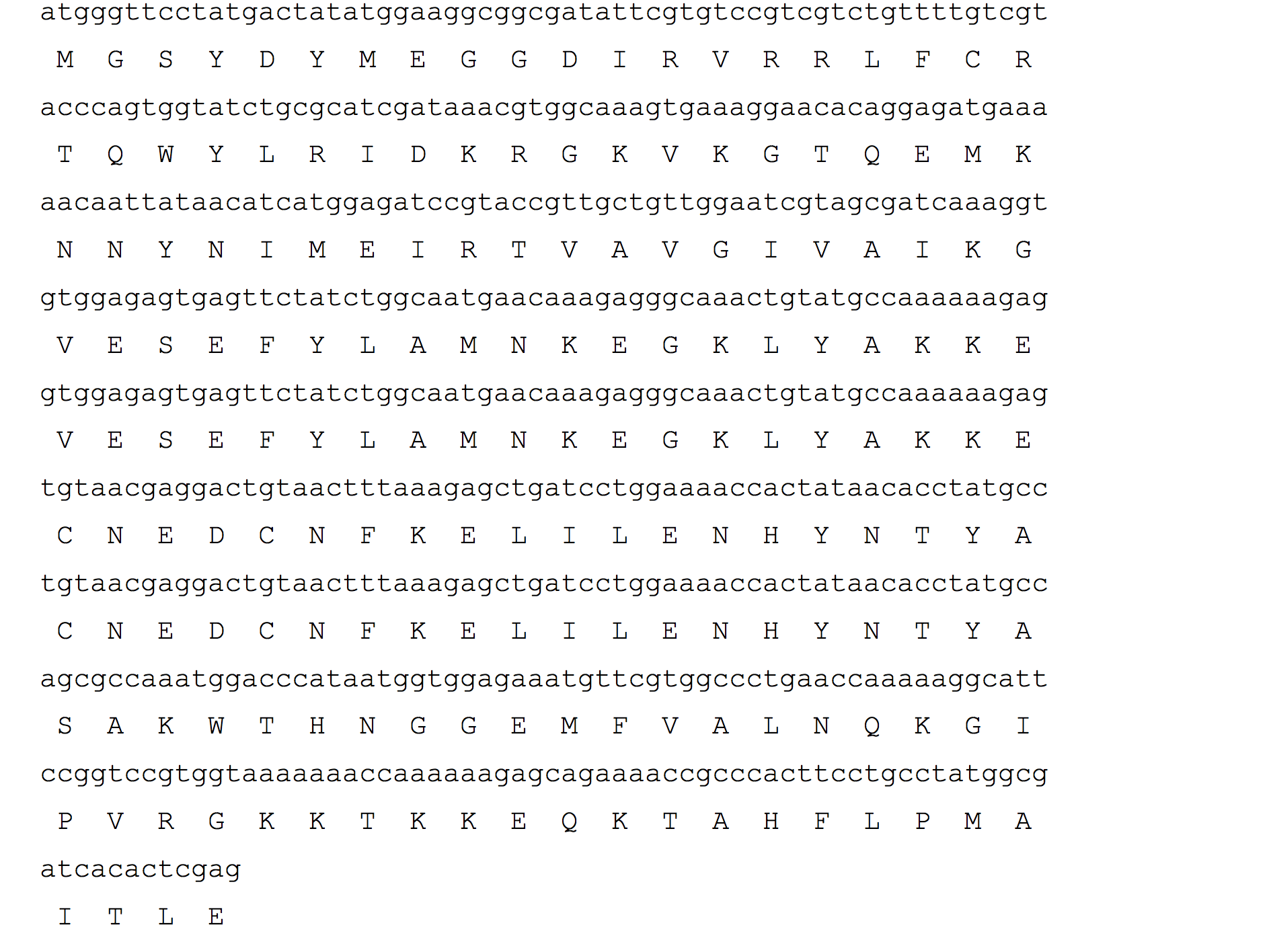

Supplement: S3 Fig — (TIFF) [file pone.0127504.s003.tiff]

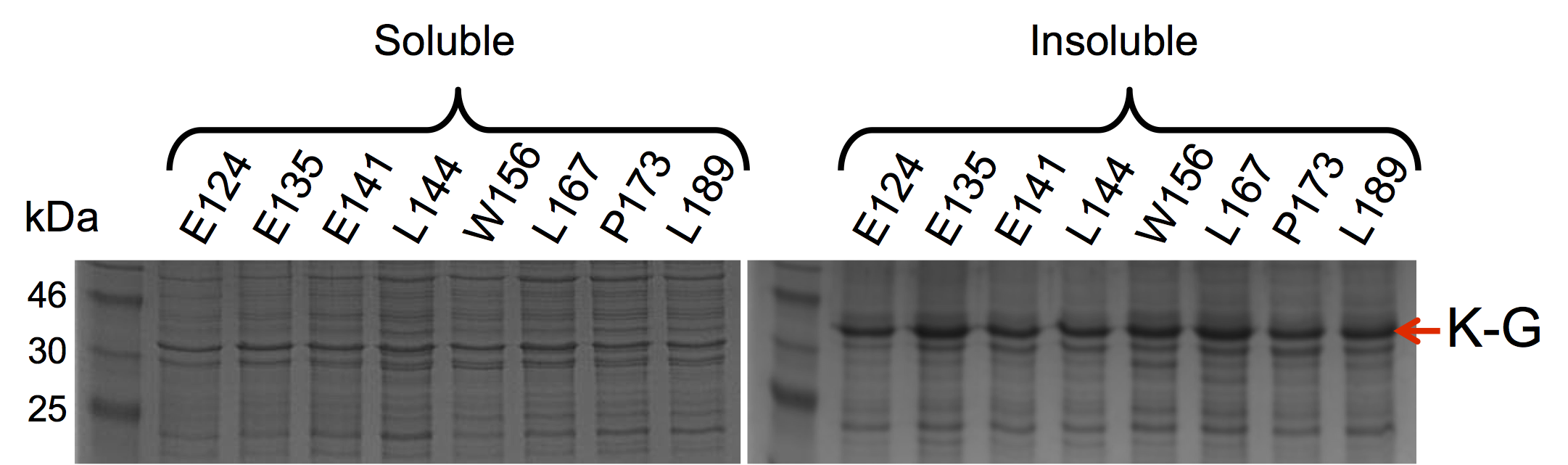

Supplement: S4 Fig — The band labelled K-G at ~44 kDa corresponds to the uncleaved KGF-sfGFP fusion product. All samples were standardised to set cell density (OD at 600 nm of 1.0). (TIFF) [file pone.0127504.s004.tiff]
