# Supplementary material for: In-Frame Amber Stop Codon Replacement Mutagenesis for the Directed Evolution of Proteins Containing Non-Canonical Amino Acids: Identification of Residues Open to Bio-Orthogonal Modification
Source: PLoS One. 2015 May 26;10(5):e0127504. doi: 10.1371/journal.pone.0127504 (PMC4444182; doi:10.1371/journal.pone.0127504)
Supplement: S1 Table — (DOCX) [file pone.0127504.s005.docx]

**S1 Table.** Oligonucleotide primer sequences.

| **Primer ID** | **Sequence (5’ to 3’** |
| --- | --- |
| 092-F | gacagcccggaaatgaaagatttc |
| 093-R | cggtgaCtAatcttcgagcttcgg |
| 102-R | cggtgCtAtatcttcgagcttcgg |
| 103-R | cggtCTAttatcttcgagcttcgg |
| 104-F | aaactcgagGGTgaaaacctgtacttccagggcagcaaaggcgaagaactgtttacc |
| 105-R | accacaGTCGACttatttatacagttcatccatgccatgg |
| 109-F | gcgtcgCATATGgcagatcttgaagacaatatggaaacc |
| 111-R | tctCTCGAGacgatacttctggtgataggcgttg |
| 112-R | gaagtacaggttttcaccctcgagacgatacttctggtgataggcgttg |
| 131-F | ttaaCCATGgcagatcttgaagacaatatggaaacc |
